# Fog-Based Two-Phase Event Monitoring and Data Gathering in Vehicular Sensor Networks

**DOI:** 10.3390/s18010082

**Published:** 2017-12-29

**Authors:** Yongxuan Lai, Fan Yang, Jinsong Su, Qifeng Zhou, Tian Wang, Lu Zhang, Yifan Xu

**Affiliations:** 1 School of Software, Xiamen University, 422 Siming South Road, Siming District, Xiamen 360000, China; laiyx@xmu.edu.cn (Y.L.); xmuzhanglu@stu.xmu.edu.cn (L.Z.); xuyifan@stu.xmu.edu.cn (Y.X.); 2 Department of Automation, Xiamen University, 422 Siming South Road, Siming District, Xiamen 360000, China; yang@xmu.edu.cn (F.Y.); zhouqf@xmu.edu.cn (Q.Z.); 3 College of Computer Science and Technology, Huaqiao University, Xiamen 360000, China; wangtian@hqu.edu.cn

**Keywords:** event detection, data-gathering, mobile sensing, VANETs

## Abstract

Vehicular nodes are equipped with more and more sensing units, and a large amount of sensing data is generated. Recently, more and more research considers cooperative urban sensing as the heart of intelligent and green city traffic management. The key components of the platform will be a combination of a pervasive vehicular sensing system, as well as a central control and analysis system, where data-gathering is a fundamental component. However, the data-gathering and monitoring are also challenging issues in vehicular sensor networks because of the large amount of data and the dynamic nature of the network. In this paper, we propose an efficient continuous event-monitoring and data-gathering framework based on fog nodes in vehicular sensor networks. A fog-based two-level threshold strategy is adopted to suppress unnecessary data upload and transmissions. In the monitoring phase, nodes sense the environment in low cost sensing mode and generate sensed data. When the probability of the event is high and exceeds some threshold, nodes transfer to the event-checking phase, and some nodes would be selected to transfer to the deep sensing mode to generate more accurate data of the environment. Furthermore, it adaptively adjusts the threshold to upload a suitable amount of data for decision making, while at the same time suppressing unnecessary message transmissions. Simulation results showed that the proposed scheme could reduce more than 84 percent of the data transmissions compared with other existing algorithms, while it detects the events and gathers the event data.

## 1. Introduction

With the development of vehicular and communication technologies, there emerges a new technology called vehicular ad hoc networks (VANETs) that integrates the capabilities of new generation wireless networks to vehicles [1,2,3]. The IEEE 802 committee defined wireless communication standard IEEE 802.11p [4], which serves specifically vehicle to infrastructure (V2I) communication. It can realize a fast V2I wireless communication connection in the urban road environment and ensure transportation safety and communication reliability for moving vehicles. The Federal Communications Commission (FCC) has allocated 75 MHz of bandwidth, which operates at 5.9 GHz for short range communications. Vehicles communicate with other vehicles, directly forming vehicle to vehicle communication (V2V), or communicate with fixed equipment next to the road, referred to as road side unit (RSU), forming vehicle to infrastructure communication (V2I). VANETs enable the concept of smart cars and intelligent transportation systems (ITS), in which information and communication technologies are applied in the fields of road transportation, traffic management and mobility management. The major goals of these activities are to increase road safety and transportation efficiency, as well as to reduce the impact of transportation on the environment. Example VANET applications include intersection collision avoidance, electronic brake lights, platooning and traffic information systems [5].

One key and challenging issue in VANETs is vehicular sensing and data-gathering [6,7,8]. Vehicular nodes are equipped with more and more sensing units, and large amounts of sensing data such as GPS locations, speeds and video clips are generated [9]. These data are shared or uploaded as input for applications aiming at more intelligent transportation, emergency response and reducing pollution and fuel consumption. In other words, cooperative urban sensing is at the heart of the intelligent and green city traffic management. The key components of the platform will be a combination of a pervasive vehicular sensing system, as well as a central control and analysis system, where data-gathering is a fundamental component that bridges the two systems. However, data-gathering and event-monitoring are also challenging issues in VANETs. VANETs differ from other mobile ad hoc networks (MANET) by their own characteristics. The network scale could be large, and the nodes (vehicles) have high computational ability and no power constraints compared to traditional sensor nodes powered by battery. As indicated by a report from Intel [10], the vehicle is evolving into a supercomputer on wheels. Furthermore, nodes are limited to road topology while moving, and the network usually suffers rapid topology and density changes under various road conditions and high moving speed. The communications are usually fragmented and intermittent-connected.

With the rapid development of embedded hardware and communication technologies, there emerges a new kind of node called fog nodes [2,11,12], which are deployed at the edge of networks and with the support of location, distribution, scalability, density of devices and mobility. The concept of fog computing extends the traditional cloud computing paradigm to the edge of VANETs. For example, Intel’s Next Unit of Computing (NUC) is a small-form-factor computer, whose motherboard measures 4 × 4 inches and could be integrated with the RSUs. Therefore, one potential solution to efficient sensing and data-gathering in VANETs is through fog nodes. The fog nodes, e.g., RSUs, are able to provide computation, storage and networking services between the vehicular nodes and ITS central servers, which brings about the opportunity for optimizing the data-gathering and event-detection processes in VANETs. The challenge of fog computing lies in that the nodes are ubiquitously connected at the edge of the network, and there should be a careful design of scheduling and cooperation schemes among the fog nodes, as well as collaboration between vehicular nodes and the cloud [13]. For the event detection applications, there is a large amount of vehicular sensed data, and they are characterized as continuous generation. The sensed data should be filtered and preprocessed before being shared or uploaded [14]. Cooperative event-driven data-filtering technologies tailored to the VANET environment are highly needed.

In this paper, we propose an efficient continuous event-monitoring and data-gathering framework called TPEG (Two-Phase Event-monitoring and data-Gathering) based on fog nodes in VANETs. TPEG would initiate an event-checking procedure to evaluate the received data. More detailed data would be uploaded to the base station when it considers this necessary, so the scheme suppresses unnecessary message transmission as much as possible while still detecting most of the events. There are two phases in the proposed scheme. In the monitoring phase, nodes sense the environment in low cost sensing mode and generate sensed data. A local fog-based two-level threshold strategy is adopted to suppress unnecessary data upload and transmission. When the probability of event is higher than the threshold, nodes transfer to event-checking phase, and some nodes would be selected to transfer to deep sensing to sense more accurate data of the environment. In the data upload phase, detailed data are uploaded to the ITS server for the final event decision. TPEG adaptively adjusts the threshold to upload a suitable amount of data for decision making, while at the same time suppressing unnecessary message transmissions. The main contributions of this paper are as follows:

1. By integrating the concept of fog nodes and VANETs, we propose an efficient scheme to efficiently monitor the events and gather data based on VANETs. The sensing operators are roughly classified into the low cost sensing (LCS) mode and the high cost sensing (HCS) mode, and by taking full advantage of the fog nodes, our scheme strikes a good balance between these two modes to achieve better efficiency;
2. The “two-level threshold adjustment” (2LTA) is proposed to avoid unnecessary event-checking and data upload. At the node level, only readings with a larger weight are sent to the RSU for further processing. The RSU then checks the confidence/probability of an event and initiates an event-checking procedure when the confidence exceeds a threshold. No event-checking procedure is needed if the confidence is within the range of thresholds;
3. Extensive experiments were conducted to demonstrate the effectiveness of the proposed algorithm. TPEG reduces more than 84% of data transmissions compared to other algorithms, while at the same time, it detects the events and gathers the event data.

The rest of the paper is structured as follows: Section 2 describes the related work; Section 3 introduces some preliminaries and defines the network model and metrics; Section 4 presents the detailed description of the TPEG algorithm, including monitoring, event-checking, data upload and threshold adjustment; Section 5 describes the environmental setup and analyzes the simulation results; finally, Section 6 concludes the paper.

## 2. Related Work

Vehicles could be viewed as powerful mobile sensors. In this section, we briefly survey some related works on data/event gathering and fog computing in VANETs.

### 2.1. Event Monitoring and Data Gathering

Lee et al. [15] proposed the MobEyes system for proactive urban monitoring. The system exploits the vehicle mobility to opportunistically diffuse concise summaries of the sensed data, and it harvests these summaries and builds a low-cost distributed index of the stored data to support various applications. Hull et al. [16] proposed a data management system called CarTelfor querying and collecting data from mobile vehicles, which enables the application development with the data collected. Palazzi et al. [17] proposed a delay-bounded vehicular data-gathering approach, which exploits the time interval to harvest data from the region of interest satisfying specified time constraints and properly alternates the data muling and multi-hop forwarding strategies. Yet, the solution has to be integrated with a time-stable geocast protocol for the query propagation and result collection. They depend on multi-hop in-network communications and “store-carry-forward” opportunistic communications, which encounter larger delay and communication cost.

Besides minimizing the delay from source to destination, event-monitoring and data-gathering protocols also try to minimize the consumed resources while ensuring that the collected information meets certain maximum delay requirements. Delot et al. [18] proposed a pull-based data-gathering strategy called GeoVanet. It adopts a distributed hash table based model to identify a fixed geographical location where a mailbox is dedicated to the query, so users are able to send queries to a set of cars and find the desired information in a bounded time. PÅĆaczek [19] introduced a method of selective data collection for traffic control applications. The underlying idea is to detect the necessity of data transfers on the basis of the uncertainty determination of the traffic control decisions, and sensor data are transmitted from vehicles to the control node only at selected time moments. Lindgren et al. [20] presented the two protocols called D-Greedy and D-MinCost for traffic-monitoring in vehicular networks. They defined two operation modes, multi-hop forwarding (MF) mode and the DTN (Delay Tolerant Network) mode (DM). During MF mode, messages are forwarded using Greedy-DTN through the shortest path to the destination, while in DM mode, messages are only forwarded at intersections to keep them inside the shortest path when the current carrier moves away.

As the volume of sensed data might be large, there is also some research on reducing the volume of sensed data and the cost of gathering them. Zekri et al. [21] proposed an event-exchanging and data-gathering scheme based on the Flajolet–Martin sketches in vehicular networks. The sketches could be exchanged without loss of information and be duplicate insensitive, so it allows manipulating the same physical repository for all vehicles. Li et al. [22] proposed a cooperative storage solution in vehicular sensor networks for mobile surveillance. Nodes first capture images from links/streets and then eliminate redundant data by exchanging image tags between vehicles, and it also includes a distributed storage balancing mechanism to offload data from heavy-load nodes to light-load nodes. Recently, the compressive sensing (CS) technique was also used for data-gathering in VANETs. Xie et al. [23] proposed the CS-based sharing mechanism to enable efficient decentralized context sharing in vehicular networks. It exploits message aggregation and the sparsity of events to reduce the total number of message exchanges. The idea of data aggregation and compressive sensing is orthogonal to the proposed TPEG mechanism, and they could be embedded in our scheme to reduce the cost of message transmissions further.

The proposed scheme differs from the above-mentioned schemes in the mode of sensing for event-monitoring. When nodes work in LCS mode, only the sketched data need to be uploaded to the road side unit for event-monitoring. TPEG takes full advantage of the computing and storage resources at the fog nodes to effectively filter unrelated and redundant sensed data to reduce the overall message transmissions.

### 2.2. VANETs and Fog Computing

Within the concept of edge computing and fog computing, more and more fog nodes would be deployed at the edge of networks for various applications. Satyanarayanan et al. [24] proposed a mobility-enhanced small-scale cloud data center that is located at the edge of the Internet. A cloudlet is a trusted, resource-rich computer or cluster of computers that is well-connected to the Internet and available for use by nearby mobile devices. Sharma et al. [25] proposed a framework for coordinated processing between edge and cloud computing/processing by integrating the advantages from both platforms. The proposed framework can exploit the network-wide knowledge and historical information available at the cloud center to guide edge computing units towards satisfying various performance requirements of heterogeneous wireless IoT networks. Similarly, Tang et al. [26] proposed a hierarchical distributed fog computing architecture for big data analysis in smart cities. It distributes intelligence at the edge of a layered fog computing network. Eltoweissy et al. [27] for the first time coined the term of autonomous vehicular clouds (AVC), where a group of largely autonomous vehicles whose corporate computing, sensing, communication and physical resources can be coordinated and dynamically allocated to authorized users. Hussain et al. [28] took a step forward to broaden the idea of VANET clouds by defining a communication paradigm for VANET clouds and then put forth the potential cloud services from the VANET standpoint. Mershad and Artail [29] proposed a system where cloud computing services are hosted by vehicles that have sufficient resources to act as mobile cloud servers and vehicles could search the mobile cloud servers that are moving nearby and discover their services and resources.

The concept of VANET cloud, indeed, is highly related to the fog computing, which extends the traditional cloud computing paradigm to the edge. Edge nodes are able to provide computation, storage and networking services between the end nodes and traditional clouds. Fog reduces service latency and improves QoS, resulting in superior user experience.

Bonomi et al. [11] defined the characteristics of fog computing and its role in the Internet of Things. They emphasized the fact that fog computing brings new elements to the realm of the Internet of Things through a reduction of service latency and improvement of QoS (quality of service). Vaquero et al. [30] gave a border and integrated view of the fog, and they argued that the fog will dramatically shift many current practices at almost every layer of the IT stack, like app development, network traffic management, network/service provision, accounting, app collaboration mechanisms, etc. Stojmenovic et al. [2] elaborated previous scenarios and further expanded this concept on a series of real scenarios, such as the smart grid, smart traffic lights in vehicular networks and software-defined networks. More recently, Kai et al. [31] gave a survey about some opportunities and challenges related to the context of fog computing in VANETs; and Zeng et al. [12] proposed a three-layer framework based on a fog structure for uploading data from sensor readings to the cloud. Static sensor nodes can forward their data to their nearby mobile fog nodes, so that the data transmission latency to the cloud center can be greatly reduced. Hao et al. [13] gave a detailed description of fog computing and proposed a flexible software architecture to incorporate different design choices and user-specified polices. The challenge of fog computing lies in that the nodes are ubiquitously connected at the edge of network, and there should be a careful design of scheduling and cooperation schemes among the fog nodes, as well as collaboration between vehicular nodes and the cloud. Aazam et al. [14] presented smart gateway-based communication, along with fog computing, for the purpose of smart communication and helping lessen the burden on the cloud. Recently, Wang et al. [32] studied the cascade shifting flow (CSF) problem and examined the wireless services provided to fast-moving users on trains based on a fog computing structure. They solved the problem by limiting the maximum shifting hops of the communication flows, thereby minimizing the maximum delay while maximizing the throughput. Wang et al. [33] discussed trajectory privacy preservation issues based on the fog structure. The main idea is to store partial important data with the dummy anonymity technology to ensure physical control, and mobile users’ partial important information can be stored on a fog server to ensure better management.

## 3. Preliminaries

### 3.1. Network and Data Gathering

**Figure 1:**
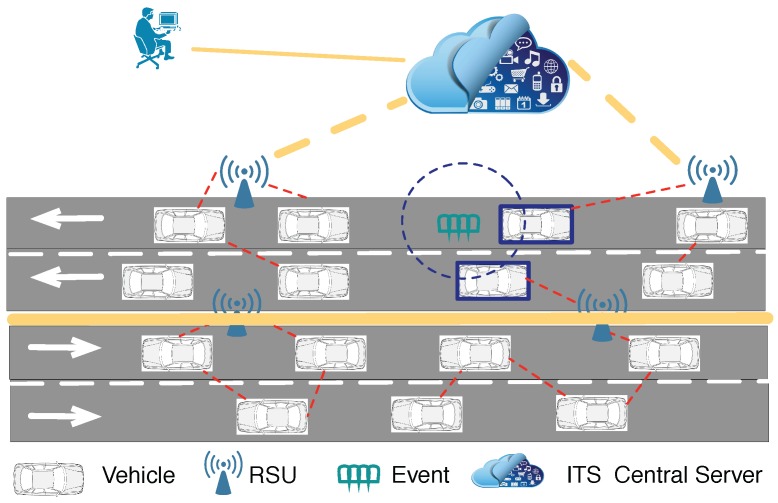
Illustration of a vehicular sensing system. Nodes generate pieces of data and send them to fog nodes (RSUs). RSUs provide computation, storage and networking services between the vehicular nodes and ITS cloud.

We assume each vehicle, $v_{i}$, monitors the road condition and surrounding environment through periodical sensing (Figure 1). It generates pieces of data and sends them to the roadside units (RSUs) through one-hop vehicle to infrastructure (V2I) or multi-hop vehicle to vehicle (V2V) transmissions. RSUs are fixed infrastructure on the roadside, which also serve as the fog nodes to provide computation, storage and networking services between the vehicular nodes and ITS (intelligent transportation system) clouds. For example, Intel’s Next Unit of Computing (NUC) is a small-form-factor computer, whose motherboard measures 4 × 4 inches and could be integrated with the RSUs. An RSU would initiate an event-checking procedure to evaluate the received data, which might lead to the upload of more detailed data to the base station when it considers this necessary. The detailed data are uploaded within a constrained time delay to the central ITS server. The server, due to its powerful computational resources and global knowledge of the road and the network, would then make the final decisions based on further data analysis.

For an event-based data-gathering process, there are three kinds of tasks:

- Continuous monitoring: the network is monitored through low-cost collection of sensing data so that ITS is informed when an event occurs. This task is represented by the event-checking procedure;
- Data gathering: more detailed data are gathered to the ITS cloud, and the data uploaded are time-limited and delay-constrained;
- Event verification: the ITS system verifies whether an event occurs and gives feedbacks to the VANETs.

Furthermore, we assume the sensing operators equipped in vehicular nodes are roughly classified into two types/modes: the low cost sensing (LCS) mode and the high cost sensing (HCS) mode. The former requires much less sensing and calculation power than the latter. Each piece of sensed data is denoted by a tuple d(id,v,α,ts,mode), where id is a unique identification of the sensing node, *v* is the sensed reading, α indicates the confidence of the true reading, ts is the timestamp and mode is the mode of sensing, which is either LCS or HCS. The HCS mode is also called deep sensing, which would generate more accurate and detailed sensing data, e.g., through more accurate and powerful sensing devises or through a higher sensing rate.

### 3.2. Event and Weight of Data

The event of interest under monitoring is denoted as *e*, and the vehicular nodes near the event would sense the event and generate some data. There is a mapping of correlation between a data reading *d* and event *e*. The correlation is indicated by function ħ(d,e):

(1) ħ(d,e)={p(e),α},p(e)∈{1,−1},α∈[0,1]

p(e) is the prediction of the event, where one denotes the event occurs and −1 denotes otherwise; α∈[0,1] is the confidence of the prediction. In this research, we aim to provide a general-purposed event-monitoring and data-gathering scheme that is to decrease the cost while at the same time achieving an acceptable detection rate. While there are different types of events, e.g., polluted air, broken road, and traffic accident, which could be detected through various vehicle-equipped sensing devices such as camera, oxygen sensor, variable reluctance sensor, etc., here, we assume machine learning algorithms are run at the vehicular and fog nodes to predict the occurrence of those events through local data. An example about the event of pavement surface deformation is given as follows:

**Example** **1.** Suppose the event e is a pavement surface deformation at location L. When Vehicle A travels though L, it senses a data reading $d_{1}$ and makes a prediction based on that reading: e happens, and the confidence is 0.5. The event systems would need more predictions like A to confirm whether e happens. Therefore, if other vehicles such as B, C, D, E also predict that e happens with large confidence, i.e., pavement surface deforms at location L with a confidence larger than 0.4, then the event detection system at fog nodes would predicate the event to be true.

Usually, the algorithms, e.g., the logistic regression [34], would indicate the event to be true or false by giving a confidence. Additionally, the weight of *d* on event *e* could be further defined as:

(2) w(d,e)=p(e)×α

The weight is highly related to the sensing operator and the monitoring applications. If the weight of data, e.g., w(d,e) is close to one, *e* is likely to occur; if it is close to −1, *e* is unlikely to occur; if it is close to zero, the occurrence of the event is unclear due to a lack of confidence. Given the type of sensing operator and data, the weight of data is used as the main input for the event-monitoring and detection. Furthermore, we assume the data gathered in HCS mode have a much larger weight than that in LCS mode:

(3) $$w\left(d_{1},e\right)<<w\left(d_{2},e\right),\;\;\;d_{1}.mode=LCS,d_{2}.mode=HCS$$

In other words, data generated through HCS mode contribute more to the ITS system to determine whether an event occurs, yet the data generated in HCS mode have also == larger sizes and incur larger sensing and gathering cost. The proposed TPEG scheme is to continuously monitor and detect events in VANETs, while at the same time reducing unnecessary high cost sensing activities, e.g., working in HCS mode, and message transmissions as much as possible.

## 4. TPEG Framework

### 4.1. Overview

**Figure 2:**
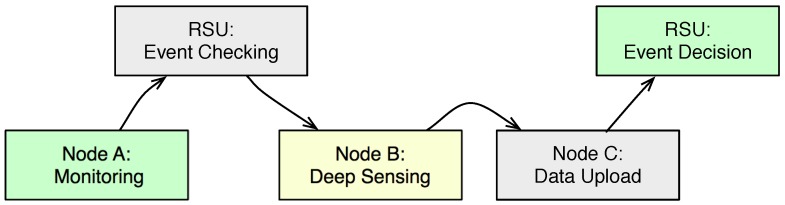
Phases of TPEG.

Figure 2 depicts the overall phases of the TPEG scheme. There are five steps as follows:

1. Data monitoring: Nodes work in LCS mode, and they sense the environment and generate the data. The data have a relatively low generation rate and confidence and are uploaded to the RSU through V2I or V2V communications.
2. Event checking: RSU checks the confidence/probability of an event, and it initiates an event-checking procedure when the event probability is high. When the confidence is low, nodes just keep silent, and no event-checking procedures are needed.
3. Deep sensing: Some nodes are selected to transfer to “deep sensing”, where they work in HCS mode and sense more accurate data about the environment.
4. Data upload: The data generated in HCS mode are uploaded to the RSU for final event verification. Data could be uploaded directly, forwarded to neighboring nodes or wait until encountering a new RSU. Nodes would adaptively decide their strategy for data upload based on whether they are within the coverage of an RSU or encountered nodes.
5. Event decision: The ITS cloud process the gathered data and make a decision about the events. Some data are archived on the cloud, and some feedback is sent back to the RSUs.

The monitoring procedure in Step 1 runs as a background process with low cost, while still being able to alert the possible events. TPEG would upload the data of the event at the deep sensing step, while suppressing the message communications at the monitoring step. It is worth being noted that as nodes are moving along the road in VANETs, TPEG is a distributed framework where the monitoring and deep sensing procedures might execute at different vehicular nodes or at different RSUs.

In the following subsections, we present the detailed description of the main steps of the scheme and algorithm.

### 4.2. Low Cost Monitoring

For an event-monitoring and detection application, each node senses the data according to the monitoring command when it moves within the area of RSU. Each node works in LCS mode and generates data. The weight of data *d* on event *e* is w(d,e), and *d* is reported to the RSU if its weight is larger than a predefined threshold:

(4) $$w\left(d,e\right)\geq \tau_{0}$$

As mentioned in Section 3, we assume the weight of data to be mainly decided by their corresponding cost, where data with lower cost have smaller weight on detecting an event. Example 2 shows low cost monitoring of the pavement surface deformation example:

**Example** **2.** By default, vehicles takes photos of the road at 300×300 px every 30 s and make a prediction and determine the confidence about the event of pavement surface deformation at different positions. Lower resolution photos incur less sensing and transmission cost, yet are less effective for the event detection compared with those of higher resolution. These low level photos might trigger the gathering of detailed data, i.e., 1080 ×1080 px per 5 s, for further analysis by the image-understanding systems [35].

### 4.3. Event Checking and Node Selection

Vehicular nodes send their readings to the RSU, so the RSU is able to calculate the weight of the received readings. For a set of readings *D*, the weight is defined as w(D):

(5) $$w\left(D\right)=\frac{\sum_{d\in D}w(d)}{\left|D\right|}$$

Given event *e*, the weight of data w(D,e) is denoted as w(D) for simplicity. If the weight for a specific event within the time window is greater than threshold $\tau_{1}$:

(6) $$w\left(D\right)\geq \tau_{1}$$

the RSU would broadcast an event-checking procedure to all nodes within its covered area. Here, we adopt a threshold-based event-checking strategy. If a threshold is broken, this means an event is likely to occur on the road, and further checking is required. Threshold $\tau_{0}$ and $\tau_{1}$ are two key parameters for our algorithm, and they strike a balance between the number of uploaded readings and the cost of event-checking procedures. In Section 4.5, we will further discuss the setting and adjustment of the parameters.

When an RSU initiates an event-checking procedure, it periodically broadcasts an “event-checking command” within its covered area, where some nodes passing by would transfer to HCS mode. However, working in HCS mode is expensive. It needs more computing and energy resources to sense more accurate data and also costs more in transmissions to upload the data. Therefore, it is crucial for TPEG to selectively pick up a part of the nodes passing-by to work on this mode.

A node creates a timer when it receives an event-checking command from an RSU for the first time. When the timer is fired, the node transfers to HCS mode. The timer is denoted tm(β), where β is the delayed interval and defined as follows:

(7) $$\beta =\left\{\begin{array}{c} BI*\frac{\tilde{t}}{\tilde{t}-t},\;\;\;t\leq \tilde{t}\;\;\\ BI*\delta ,\;\;\;\;t>\tilde{t}\;\; \end{array}\right.BI<\tilde{t},\;\;\delta \in \left(0,1\right)$$

where BI denotes the time interval of the broadcasting of event-checking messages at RSU, $\tilde{t}$ is the average duration of a node moving through the RSU coverage area, *t* is the elapsed time when a node enters the coverage area and δ is a random number. The average duration $\tilde{t}$ could be derived by the historical duration of nodes passing by a specific RSU. Nodes enter the RSU covered area in sequential order, and the node that newly enter the area would have the smallest delay interval according to Equation (7). It would have its timer tm fired and then accept the command of working in HCS mode with less delay. Furthermore, the broadcasting interval BI is set to be smaller than the average pass duration $\tilde{t}$, so when a node goes through the RSU coverage area, it would receive at least one broadcasting message. In extreme cases when the elapsed time is greater than $\tilde{t}$, e.g., traffic jam, the delay β would be randomly set, and nodes are randomly selected to work in HCS mode. The interval of the timer is set according to BI, and it could be tuned to make the timer smaller to avoid the case that vehicles leave the coverage area of an RSU without vehicles in HCS mode. Moreover, we are assuming a urban scenario where vehicles do no travel so fast.

If a node accepts the event-checking command, it would transfer to HCS mode and send an accept-HCS message to the RSU, where other nodes overhearing this message would cancel their timers. Therefore, only a small part of the nodes would join the event-checking procedure, and some nodes would still work in LCS mode. For the RSU, it periodically broadcasts the event-checking command if it does not receive an accept-HCS command from passing-by nodes. When it receives the accept-HCS message, it would broadcast an on-checking command, and other nodes that receive this message would cancel their timers for accepting the event-checking command.

### 4.4. Adaptive Data Upload

Nodes that accept the event-checking command would transfer to deep sensing for a period of time, and a relatively larger size of data would be generated in HCS mode. The data are uploaded to the RSUs and then routed to the ITS system for event analysis as RSUs are inter-connected through wired networks or the Internet.

A node would schedule its data for upload when it is within the coverage of an RSU. When it moves out of the RSU coverage, it would delay the data upload until the next opportunity of entering an RSU, or forward the data to an encountered vehicular node. For the ease of data upload and transmission, a piece of HCS data would be split into small segments. For a data segment, e.g., ds, there are three cases for it to be uploaded to the ITS system:

1. When the node is within the coverage area of RSU, ds is directly scheduled for uploading, which is denoted as:
  (8) method(ds)=Upload,ifrsu(s)=true;
  where *s* is the node that holds ds and rsu(s) returns true if node *s* is within the coverage of an RSU.
2. When there is no RSU coverage or node-to-node connections, ds is stored locally, and the upload is suppressed, which is denoted as:
  (9) method(ds)=Delay,ifrsu(s)=false;&neighbor(s)=ϕ
  where neighbor(s) denotes the set of neighbors of node *s*.
3. When a node moves out of the RSU coverage and encounters a neighboring node, it would decide its forwarding strategy based on the current node *s* and the encountered node $s^{\prime }$. ds might be forwarded to $s^{\prime }$ or be stored at *s* and waits for other transmission opportunities. This is denoted as:
  (10) $$method\left(ds\right)=\left\{\begin{array}{c} Forward,\;\;\;\mathrm{if}\;\;fw\left(s,s^{\prime }\right)=true\;\&\;\;rsu\left(s\right)=false\\ Suppress,\;\;\;\mathrm{if}\;\;fw\left(s,s^{\prime }\right)=false\;\&\;\;rsu\left(s\right)=false \end{array}\right.$$
  where $fw(s,s^{\prime })$ denotes the feasibility of forwarding data from node *s* to $s^{\prime }$. $fw(s,s^{\prime })$ returns true if the data are forwarded to $s^{\prime }$, and node $s^{\prime }$ would go into an RSU coverage area and upload the data before a predefined deadline. In more detail, $fw(s,s^{\prime })$ is true if it satisfies the following condition:
  (11) $$et\left(s^{\prime }\right)+ut\left(ds\right)\leq TC<et\left(s\right),\;\;\;ut\left(ds\right)<cd\left(s,s^{\prime }\right)$$
  Here, TC is the time constraint of data to be uploaded to the ITS system, $et(s^{\prime })$ is the expected time interval for node $s^{\prime }$ to enter an RSU coverage area and ut(ds) is the time duration of data uploading, which could be easily calculated by dividing the data size by the bandwidth: $ut\left(ds\right)=\frac{\left|ds\right|}{bw}$. $cd(s,s^{\prime })$ is the expected contact duration of node *s* and $s^{\prime }$.

Here, we borrow the idea of “store-carry-forward” in delay-tolerant networks [36] for the data uploading and forwarding. When a node, e.g., $s^{\prime }$ receives data from another node, $s^{\prime }$ is responsible for the data uploading. $s^{\prime }$ might upload the data when it is within an RSU’s coverage, or forward it to another node based on Equation (11). The calculation is mainly based on the expected time interval to reenter an RSU coverage area, as well as the expected contact duration between two encountered nodes, which are learned from the network meta-data and historical data. Each node would predict its time left to the next RSU (et(s)) based on their routes or historical trajectories, as well as contact histories with RSUs. The expected contact duration ($cd(s,s^{\prime })$) between two encountered nodes could also be estimated based on their direction, speed and trajectory records. These metadata and parameters are common input for the routing protocols for VANETs. Readers could refer to [2] for further discussions.

### 4.5. Threshold Adjustment

When an RSU initiates an event-checking procedure, more detailed and accurate sensed data would be uploaded and gathered. These data are processed by the central ITS system, and the final decision about the events would be made either by more intelligent analysis or human intervention. There are two aspects where TPEG can reduce unnecessary message transmissions:

- Events are assumed to be uncommon phenomenon. When events do not occur, the network should avoid unnecessary event-checking procedures.
- Unlike some monitoring and detection scenarios where events are transient, events at VANETs would usually last for a period of time. Therefore, when an event occurs, it is important for nodes not to be triggered by HCS mode and not to upload the redundant data to the RSU.

The pavement surface deformation example belongs to the permanent monitoring scenarios because it would need some time (days or weeks) for road administrations to repair them. Car accidents such as slight car crashes belong to the transient monitoring case. Usually, the accident would be handled in several minutes. TPEG adopts a mechanism called “two-level threshold adjustment” (2LTA) to avoid unnecessary event-checking and data upload. 2LTA adaptively adjusts the thresholds at the node and the RSU levels to suppress unnecessary sensing and data upload operations, while at the same time detecting as many events as possible.

#### 4.5.1. Node Level Adjustment

At the node level, ordinary nodes send monitoring readings to RSUs so that an event-checking procedure would be triggered. Readings with a weight larger than $\tau_{o}$ are sent to the RSU for further processing. Each node moving through the RSU coverage area would send a histogram-based sketch about its monitored readings to the RSU. A sketch sk is composed of tuples in the form:

(12) $$\left\{<\left(h_{1},h_{2}\right],n_{1}>,..,<\left(h_{i},h_{i+1}\right],n_{i}>,..,<\left(h_{k-1},h_{k}\right],n_{k}>\right\}$$

where $\left(h_{i},h_{i+1}\right]$ is the range of weight and $n_{i}$ is the number of readings whose weight is within the range. Sketches could be stored in any histogram like data structures. Suppose RSU expects to receive *m* readings for the event-checking decision, then the threshold $\tau_{o}$ is set as follows:

(13) $$\tau_{0}=argmin_{x}f\left(x\right)=\left\{\sum_{sk\in \Phi }\eta \left(sk,x\right)\geq m\right\},\;\;x\in \left\{h_{0},h_{1},..,h_{k-1}\right\}$$

where η(sk,x) is the number of readings in sketch sk whose weight is larger than *x*, Φ is the set of sketches received at the RSU during the average duration of a node moving through the RSU. The node receiving the update message from the RSU would just reset the threshold $\tau_{0}$.

#### 4.5.2. RSU Level Adjustment

At the RSU level, $\tau_{1}$ is adjusted according to the event detection result (Algorithm 2, Lines 14–16). If the event is verified to be false (false positive events) after an event-checking procedure, TPEG would adjust $\tau_{1}$ to be a little larger:

(14) $$\tau_{1}=\tau_{1}\times \left(1+\Delta \right)$$

where Δ is a predefined increment factor, e.g., Δ=0.05 by default. Yet, if there are several events that are verified to be false, $\tau_{1}$ might be adjusted to a larger value, and triggering the event-checking procedure would be hard. To avoid this drawback, $\tau_{1}$ would also be set a little smaller with the time of epochs passed by:

(15) $$\tau_{1}=\tau_{1}\times \left(1-\Delta \times \frac{tu}{TU}\right)$$

where tu is the time interval since the latest $\tau_{1}$ adjustment and TU is the unit of time for the threshold adjustment, which is predefined.

When the cloud receives the sensing data from the RSUs, it would store the data, check if the event happened and send the checking results to the RSU. If an event is verified to be true after the event-checking procedure, it is registered at the ITS system, and actions would be taken to handle the event. For example, events such as “bad road condition” or “road blocks” would be handled by the municipal administration after a period of time. Yet, during this period of time, the RSU would decrease the chance of initiating event-checking procedures. It periodically broadcasts an event-occur message to alert vehicular nodes who newly enter its coverage area, and nodes that receive the event-occur message would keep working in LCS mode to save redundant sensing operations and message transmissions. When events are handled, they are removed from the ITS system, and the RSU would broadcast an event-removed message to notify ordinary nodes to transfer back to normal monitoring and event detection tasks.

### 4.6. Algorithm Descriptions

Algorithms 1 and 2 are the pseudocodes of the message handling at ordinary nodes, RSUs and the cloud, which illustrate the procedures of the TPEG scheme. For an event-monitoring and detection application, each ordinary node works in LCS mode and generates data (Algorithm 1, Line 1). The weight of data *d* on event *e* is w(d,e), and *d* will be reported to the RSU if its weight is larger than a predefined threshold $\tau_{0}$ (Algorithm 1, Lines 2–3): When the RSU receives the LCS data from ordinary nodes, it stores the data and calculates the weight of the event based on the accumulated dataset (Algorithm 2, Lines 2–3). If the weight of the event exceeds the threshold $\tau_{1}$, the RSU initiates an event-checking procedure (Algorithm 2: Lines 4–5). It broadcasts the event-checking command within its coverage and waits to receive the HCS messages (Algorithm 2, Lines 6–7). The command is then received by ordinary nodes. An ordinary node would create a timer tm(β) when it receives an event-checking command from an RSU for the first time. When the timer is fired, the node transfers to HCS mode, and more detailed data would be uploaded to the RSU (Algorithm 1, Lines 4–8). When the HCS data are received by the RSU, they would be forwarded to the cloud (Algorithm 2, Line 9).

When the cloud receives the sensing data from the RSUs, it would store the data, check whether an event has happened by its backend event analysis system and send the final result, i.e., the EVENT message, back to the RSU (Algorithm 2, Lines 20–23). When an RSU receives the EVENT message, if the event is verified to be false, it would adjust its threshold $\tau_{1}$ for the event through Equation (14) (Algorithm 2, Lines 14–16). $\tau_{1}$ might be adjusted to a larger value, which makes it harder to trigger the event-checking procedure. Threshold $\tau_{1}$ is also updated by Equation (15) as time passes by (Algorithm 2, Lines 17–18).
**Algorithm 1:** Handling messages at ordinary nodes.
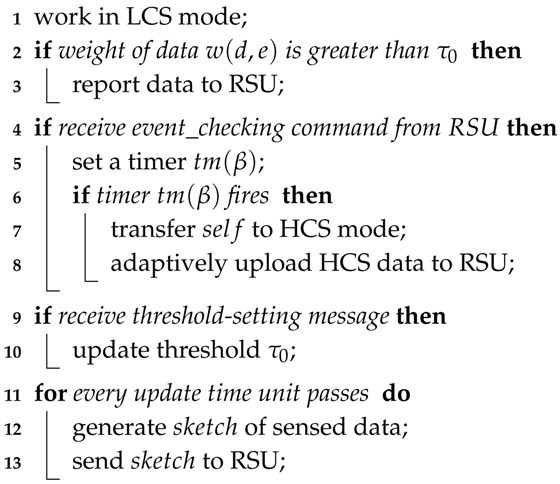

**Algorithm 2:** Handling messages at RSUs and the cloud.
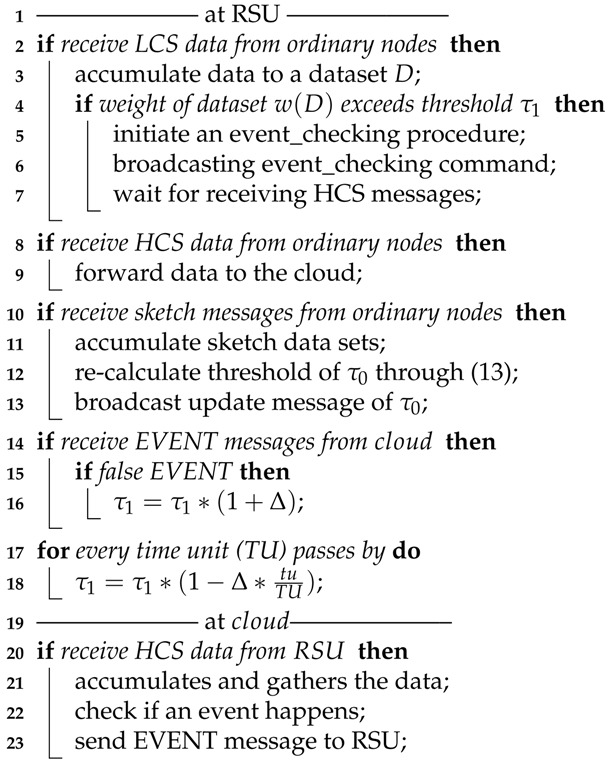


The threshold at the ordinary nodes, denoted by $\tau_{0}$, is calculated and updated through sketches. For every unit of update time, a sketch is generated at ordinary nodes and sent to the RSU (Algorithm 1, Lines 12–13), where the threshold $\tau_{0}$ is recalculated through Equation (13) (Algorithm 2, Lines 14–12). The new threshold is then broadcast through a update message within its coverage (Algorithm 2, Line 13). The node receiving the update message from the RSU would just reset the threshold $\tau_{0}$ (Algorithm 1, Line 10).

## 5. Experimental Study

### 5.1. Environmental Setup

**Figure 3:**
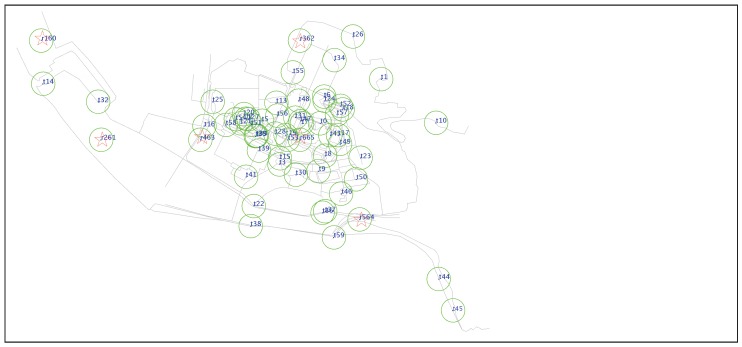
Part of the snapshot of the simulation field where six RSUs and 60 vehicular nodes are deployed at Xiamen University campus centered at latitude: 24.4390262, longitude: 118.0977218. Circles denote the nodes and their sensing areas; five-pointed stars denote the RSUs.

To verify the performance of the proposed algorithm, we conduct experiments on the ONE platform [37] with a real-world road network. ONE is a simulation environment that is capable of generating node movement using different movement models routing messages between nodes with various DTN routing algorithms. There are six fog nodes/RSUs and 60 vehicular nodes in the network, which is part of the campus of Xiamen University centered at latitude: 24.4390262, longitude: 118.0977218 (Figure 3). The total simulation time is 43,200 s. To simulate the monitoring application, events and sensing data are injected through a data generator. The interval between two sequential events follows the Poisson distribution with parameter λ = 200 s. Each event is attached to a location along the road and would be handed over or nullified after within 1000–5000 s. For event *e*, it has an impact area with a radius of $R_{e}$. When a node enters the impact area, its sensed data would reflect the impact of that event. The weight of sensed data follows the normal distribution $w_{x}\left(d,e\right)∼N\left(\alpha_{x},\sigma_{x}\right)$:

(16) $$\alpha_{x}=\left\{\begin{array}{c} \epsilon_{x},\;\;dis\left(s,e\right)>R_{e}\\ \epsilon_{x}+\xi_{x}*\frac{R_{e}-dis\left(s,e\right)}{R_{e}},\;\;dis\left(s,e\right)\leq R_{e} \end{array},\;\;\;\;x\in \left\{LCS,HCS\right\}\right.$$

where the subscript *x* could be *l* or *h* representing the LCS and HCS modes, $\epsilon_{x}$ represents the impact of sensing noise and dis(s,e) is the distance between source *s* and event *e*. $\xi_{x}$ represents the impact of the event on the sensing data, and $\frac{R_{e}-dis\left(s,e\right)}{R_{e}}$ increases when node *s* gets closer to the event, which would increase the weight of data. The average duration of nodes passing by an RSU, $\tilde{t}$, is calculated by dividing the length of the road segment that the RSU covers by the average speed of vehicles. Furthermore, the sensing frequency and data size are also defined by the mode of sensing. When a node is in LCS mode, it generates a piece of data of 50 K; when a node works in HCS mode, it generates a piece of data with 500 K. The bandwidth of the link that uploads data from ordinary nodes to RSUs is 500 K/s. We assume ideal links when two nodes meet and establish a connection. Table 1 lists the default parameters of the simulation setting.

### 5.2. Metrics and Compared Algorithms

The event detection accuracy and overall system cost are used as evaluation metrics for the evaluation of the algorithms. The accuracy could be further divided into recall rate (p1) and precision (p2), which are defined as follows:

(17) $$p1=\frac{n_{0}}{n_{0}+n_{1}};\;\;\;p2=\frac{n_{0}}{n_{0}+n_{2}}$$

where $n_{0}$ is the number of detected true events, $n_{1}$ is the number of undetected true events and $n_{2}$ is the number of detected false events. Precision (also called positive predictive value) is the fraction of relevant events among the retrieved events, while recall (also known as sensitivity) is the fraction of relevant events that have been retrieved over the total amount of relevant events.

Nodes sense the data and send them to the RSUs, and events are verified by the ITS servers. The overall cost contains the cost of sensing at local nodes, computation at nodes, RSUs and the ITS center and data transmissions among nodes and RSUs. When nodes work in HCS mode, they generate more data and hence need to upload a larger amount of data to the RSUs. For simplicity, we use the total number of messages sent as an indicator for the overall cost, and the average time spent on detecting the events is also considered. The average results of five simulations are presented in this section.

There is not much research directly related to the fog-based data-gathering and event-monitoring in VANETs. For fair comparison with the proposed algorithm, we also conducted another four data-gathering and event detection schemes: (1) NAIVE: nodes sense in HCS mode periodically all the time, and whenever a node encounters an RSU, its data are uploaded to the RSU and then forwarded to the cloud for event detection. (2) PROPHET [38]: a contact history-based protocol. Nodes exchange data according to the data forwarding probability to RSUs based on the contact history; (3) ESSMD [39]: the takes advantage of all chances to upload the data, but reduces the number of event broadcasting when vehicles detect the same event in close proximity. The PROPHET scheme is designed for the delay-tolerant network (DTN) [7], where obviously, VANETs comprise one kind of DTN. PROPHET and ESSMD are classic and provide good comparison to our scheme, which aims to decrease the cost while at the same time achieving an acceptable detection rate.

### 5.3. Overall Performance

Table 2 presents the overall performance of the algorithms. The proposed scheme could reduce more than 84 percent of the data transmissions compared with other existing algorithms, while at the same time it detects the events and gathers the event data.

About 160 events are generated, and the number of true positive, true negative, false positive and false negative events is also presented. NAIVE has the lowest message transmissions, and the recall rate of NAIVE is 0.2648, which is the lowest. This is because for NAIVE, data are uploaded only when a node encounters an RSU. The number of data transmissions is about $2.34\times 10^{4}$, and many data have to be stored locally and dropped when there is not enough storage space, which leads to some loss of event detections. Therefore, NAIVE has the lowest recall rate and precision of event detections.

For PROPHET and ESSMD, data are forwarded to encountering nodes, and all chances are utilized to upload the data. They have a much higher recall rate, which is about 0.93, and also incur a larger cost of data transmissions. The numbers of message transmissions of PROPHET and ESSMD are about $3421.98\times 10^{4}$ and $2583.36\times 10^{4}$ respectively. In contrast with other algorithms, the proposed TPEG scheme has a much lower cost of data transmissions; the number of message transmissions is about $403.26\times 10^{4}$, which is about eight percent of that of PROPHET and about 15.6 percent of that of ESSMD. This is because TPEG adopts LCS mode for event-monitoring, and detailed data are generated and uploaded only when a threshold at the RSU is broken. This generates far fewer data and incurs much lower transmission cost. The reduced transmission of messages, however, does not harm the precision and recall of the detection of events. The precision of the three algorithms is within the range of [0.97,0.99], and the recall of TPEG is 0.9226, about 0.01 lower than those of PROPHET and ESSMD. Through the two-phase threshold suppression, TPEG reduces much data transmissions compared with other algorithms, while at the same time detecting all the events and gathering the event data.

Table 2 also presents the time needed for detecting an event. If an event is generated at $t_{1}$ and is confirmed at ITS at $t_{2}$, then $|t_{2}-t_{1}|$ is the time needed for the event detection. From the table, we could see that the average detection time of NAIVE is 36.29 min, which is the longest, and those of PROPHET, ESSMD and TPEG are about 28.83, 30.6 and 33.69 min, respectively. NAIVE has to wait for some time before uploading the data when a vehicular node is not covered by the RSU, yet other schemes could forward the data to encountered vehicles, who help to upload the data for event detection. The length of the detection time of TPEG is larger than PROPHET and ESSMD because not all the nodes broadcast and forward the sensed data. For TPEG , when an “event-checking procedure” is issued, the network would select a node for the data upload and forwarding, which leads to some delay of the event detection.

### 5.4. Impact of Factors

From the overall performance analysis, we could see that TPEG has great improvement compared with other schemes on the overhead of message transmissions. The advantage lies in the two modes of sensing, as well as message suppression through thresholds. In this subsection, we vary the parameter settings and study their impacts on the performances.

**Figure 4:**
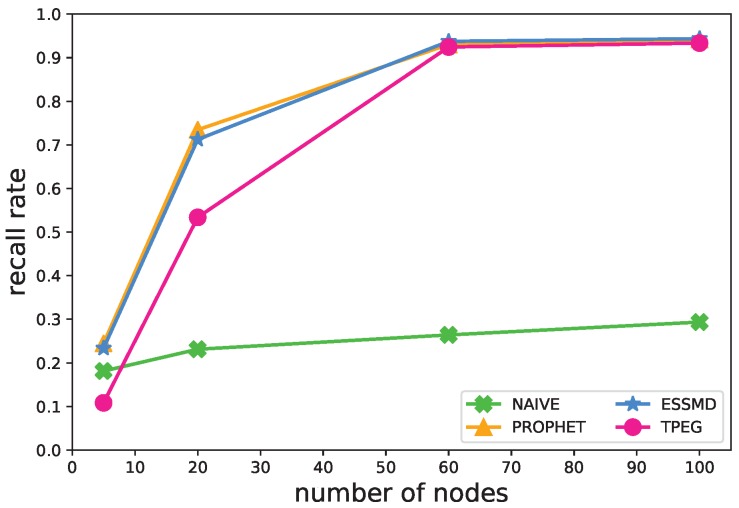
Impact of the number of nodes on the recall rate.

Figure 4 shows the impact of the number of nodes on the recall rate, which goes up with the amount of nodes within the network. When there are fewer nodes, e.g., five, many events are not detected, and the recall rate is less than 0.27. This is because there are not many vehicular nodes sensing the events, and consequentially, only a small number of samples is generated and many events missed. As mentioned previously, in the NAIVE scheme, data are uploaded only when a node encounters an RSU, so its recall rate is the lowest, and the increase in the number of nodes has less impact on the rate. When the number of nodes increases, the recall of all the schemes except NAIVE goes up quickly. The recall of TPEG is a little bit smaller than those of PROPHET and ESSMD. When there are more than 60 nodes within the network, its recall rate goes up to about 0.92, which is almost the same as those of PROPHET and ESSMD.

#### 5.4.1. Number of Nodes

**Figure 5:**
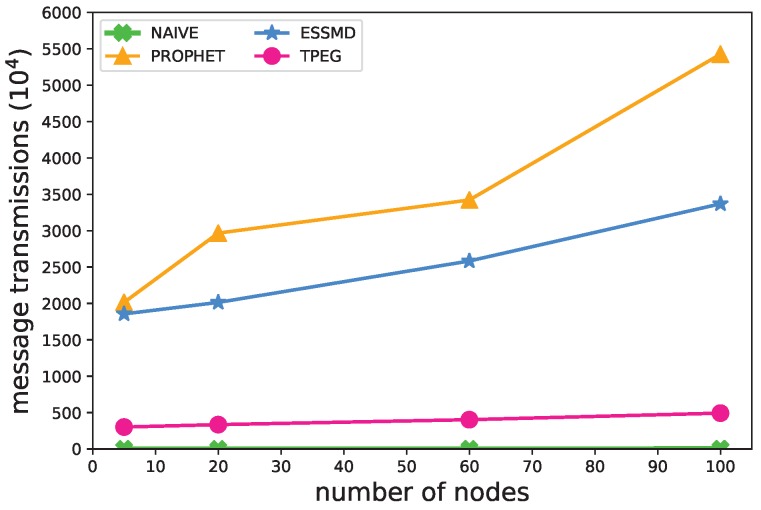
Impact of the number of nodes on the number of message transmissions.

Figure 5 shows the impact of the number of nodes on the message transmissions. When there are more nodes in the network, more messages are transmitted with the network and more data are uploaded to the RSU for all the schemes. Yet, for the PROPHET scheme, the increase of the messages is drastic because nodes would broadcast messages to their neighboring nodes. For the ESSMD scheme, it adopts a controlled broadcasting strategy, so the increase of the number of messages is smaller. For the TPEG scheme, it adopts a threshold-based strategy to suppress the data upload, which incurs fewer transmissions. The amount of messages transmitted is less than 16.2% of those of PROPHET and ESSMD. Additionally, the increase of TPEG with the number of nodes is much smaller, as shown by the flat line. Figure 5 shows that NAIVE and TPEG scale well with the number of nodes, yet TPEG has a far larger event-detection rate.

**Figure 6:**
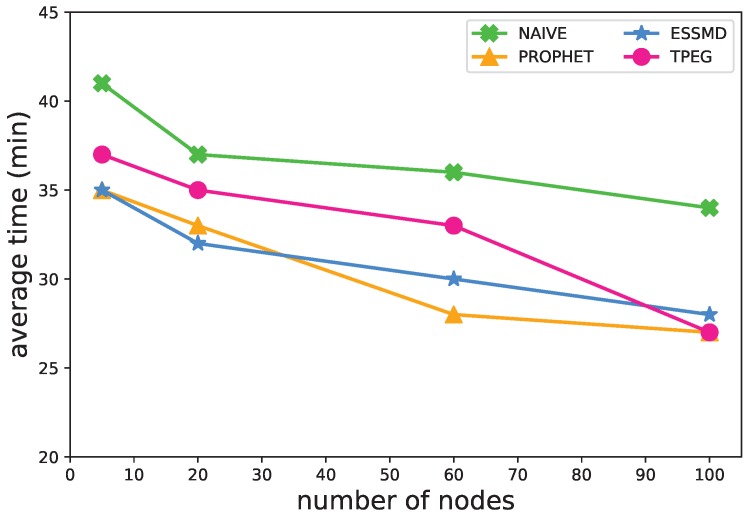
Impact of the number of nodes on the average detection time.

Figure 6 shows the impact of the number of nodes on the time needed for detecting events. The time for event detection goes down as the number of nodes increases. The more vehicular nodes moving on the roads, the more data are sensed and uploaded with less delay to the RSUs for the event detection. For example, in the proposed TPEG scheme, the average event detection time is about 37 min when there are five nodes, and about 27 min when there are 100 nodes in the network. When there are fewer than 60 nodes, the detection time is about 5–10 min longer than those of PROPHET and ESSMD. Yet, when there are more nodes on the network, e.g., 100, the detection time of TPEG is almost the same as that of PRPHET.

#### 5.4.2. Size of Cache

**Figure 7:**
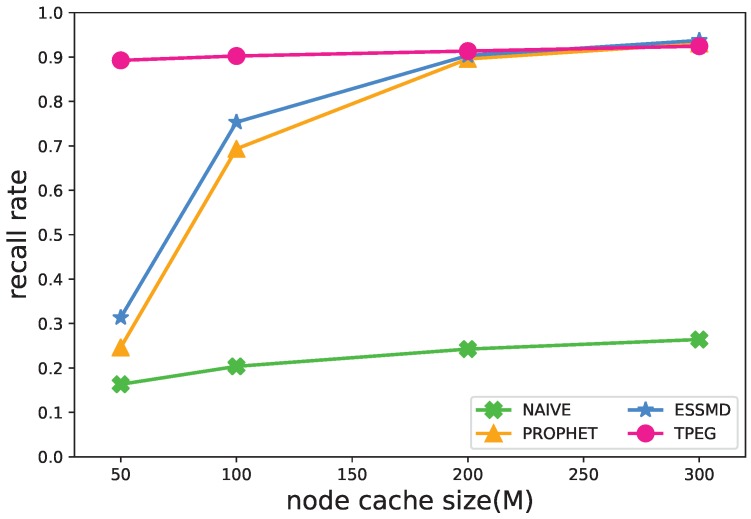
Impact of cache size on the recall rate.

**Figure 8:**
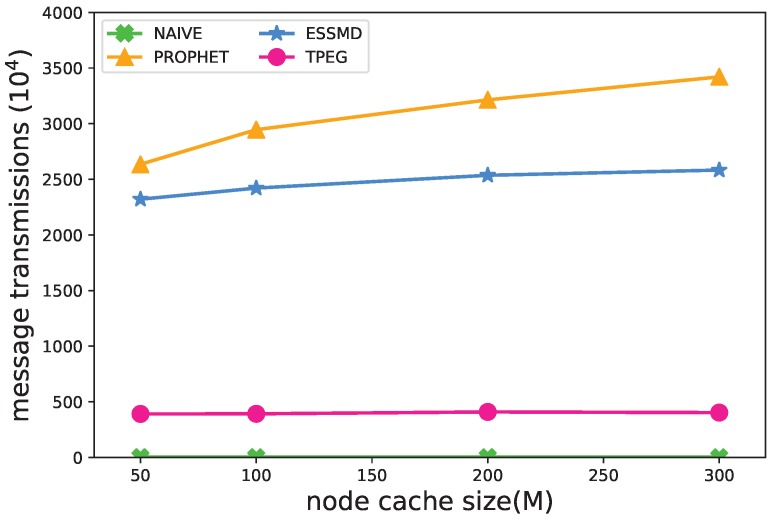
Impact of cache size on the number of message transmissions.

The vehicular nodes adopt a “store-carry-forward” strategy [40] for the message transmissions, so the cache size of nodes would have an impact on the performance. Figure 7 and Figure 8 show that the recall rate and message transmissions grow as the cache size increases. This is true especially for PROPHET and ESSMD, because within these schemes, the nodes work in the high cost sensing mode as the default, and they would diffuse the sensed data to neighboring nodes, which requires a larger cache size for the data storage, and the message transmissions would increase. When the cache size is small, e.g., 50 Megabytes, some data are dropped, and the recall of events is lower than 0.3 and the message transmissions relatively low. For the TPEG scheme, the cache size has less impact on the recall rate and the message transmissions. When the cache size grows from 50 M–300 M, the recall rate increases from 0.8926–0.9245 and the message transmissions grow from $302\times 10^{4}$–$493\times 10^{4}$. This is because the nodes generate data in LCS mode by default, which requires less cache space. Moreover, the two-level thresholds suppress the message diffusion and upload and further reduce the message transmissions.

**Figure 9:**
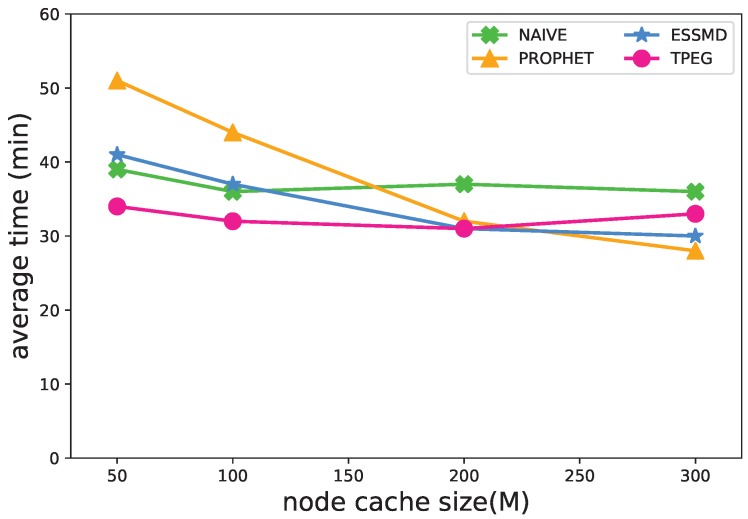
Impact of cache size on the average detection time.

Figure 9 shows the impact of cache size on the detection time. For the PROPHET and ESSMD scheme, the detection time decreases as the cache size grows. The length of time drops from 51(41)–28(30) for PROPHET(ESSMD) when the cache size grows from 50 M–300 M. This is because when there are larger cache sizes, a larger number of messages is transmitted, and more data are uploaded to RSU to speed up the detection of events. For the NAIVE and TPEG scheme, the cache size has little impact on the event detection time.

#### 5.4.3. Sensing Frequency

**Figure 10:**
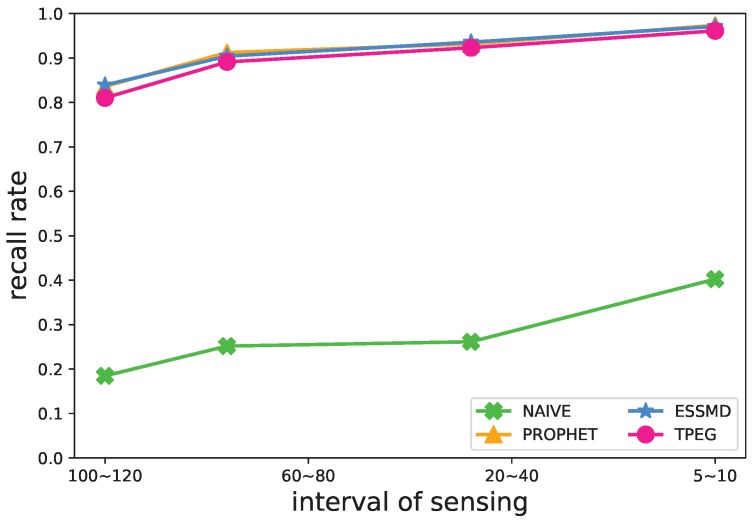
Impact of sensing interval on the recall rate.

**Figure 11:**
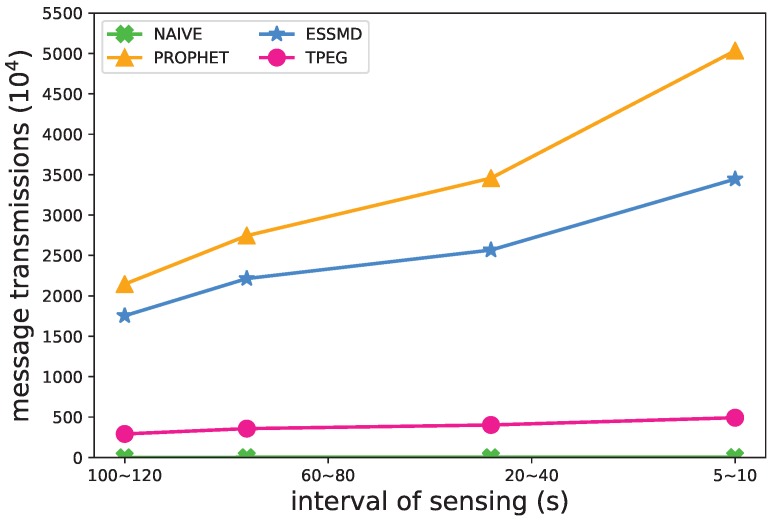
Impact of sensing interval on the number of message transmissions.

**Figure 12:**
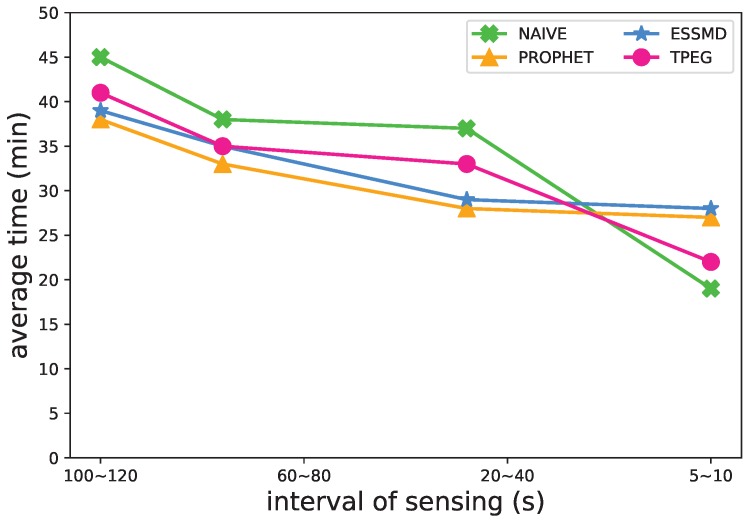
Impact of sensing interval on the average detection time.

Another impact factor of the event detection is the sensing frequency of nodes. In Figure 10, Figure 11 and Figure 12, we varied the sensing interval to study its impact on the recall rate, message transmissions and the length of detection time. When the sensing frequency increases, or the interval of sensing decreases, more sensing data are generated and uploaded to the RSUs for further processing, so the recall rate and the message transmission grow (Figure 10 and Figure 11), and the average detection time decreases (Figure 12) for all the schemes. TPEG has a similar recall rate as PROPHET and ESSMD, yet has a much lower cost of message transmissions. For all the schemes except NAIVE, the recall grows from 0.82 to 0.97 when the interval of sensing decreases from 100∼120 s to 5∼10 s. For the PROPHET and ESSMD scheme, the message transmissions grow about 1.96∼2.34-times more, while the TPEG only increases about 69 percent, from $291\times 10^{4}$ to $492\times 10^{4}$. This is largely due to the low cost of LCS monitoring mode in the TPEG scheme. The proposed scheme scales well with the sensing frequency.

#### 5.4.4. Threshold Adjustment

**Figure 13:**
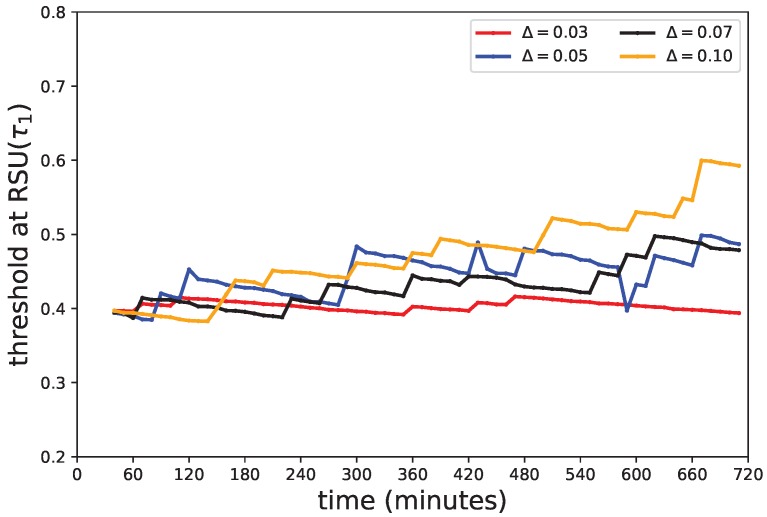
Change of threshold $\tau_{1}$ with time.

TPEG adopts the 2LTA strategy to adaptively adjust the thresholds at the node and the RSU levels to suppress sensing and data upload operations. According to Equation (13), the threshold $t_{0}$ is dynamically set according to *m*, which is the expected number of readings received by the RSU for the event-checking decision. Threshold $\tau_{1}$ would increase with the incremental factor Δ according to Equation (14) and would decrease with the time of epochs passed according to Equation (15). $\tau_{1}$ is initially set to 0.4, *m* to 3 and TU to 10,000 through parameter tuning. Figure 13 shows the change of $\tau_{1}$ with time, and Table 3 shows the impact of Δ for TPEG. When Δ is larger, e.g., 0.1, $\tau_{1}$ goes up with time more quickly and obviously. When Δ goes up from 0.03–0.1, the recall goes down from 0.9554 to 0.8929, the message transmission decreases from $618.77\times 10^{4}$ to $532.22\times 10^{4}$ and the average detection time goes up from 24.73 min to 37.55 min. This is because larger Δ leads to larger $\tau_{1}$, which leads to a lesser number of event-checking procedures, which in turn leads to a smaller amount of data transmissions and some misses and delay of event detections. When Δ is in the medial, e.g., 0.05, $\tau_{1}$ sways accordingly to time. Its recall, transmissions and detection time are 0.94, $578.38\times 10^{4}$ and 25.65, respectively, which are in the medial range of all the results.

## 6. Conclusions

In this paper, we proposed an efficient continuous event-monitoring and data-gathering scheme called TPEG based on fog nodes in VANETs. A fog-based two-level threshold strategy is adopted to suppress unnecessary data upload and transmissions. In the monitoring phase, nodes sense the environment in low cost sensing mode for data generation. These data are then sent to the RSUs, and the confidence of events is calculated. If the confidence exceeds some threshold, nodes would transfer to event-checking phase, and some nodes would be selected to transfer to deep sensing mode to sense more accurate data of the environment. TPEG adaptively adjusts the threshold to upload a suitable amount of data for decision making, while at the same time suppressing unnecessary message transmissions. Experimental studies demonstrate that the proposed scheme could reduce more than 84 percent of the data transmissions compared with other existing algorithms, while at the same time, it detects events and gathers the event data with some delays.

For future work, we are going to use real-world vehicular trajectories and log data of vehicles to demonstrate the effectiveness of the proposed scheme. Furthermore, we are going to broaden the definition of events and use the scheme as a basic event-monitoring and data-gathering block to construct specific VANET applications. 

## Figures and Tables

**Table 1:** Default parameters of the simulation. LCS, low cost sensing; HCS, high cost sensing.

Parameter	Value	Description
*n*	60	number of vehicular nodes
n_RSU	6	number of RSU nodes
n_time	43,200 s	simulation duration
L	3000 m	length of road segment
d_time	∼[25–35] s	interval of sensing
$R_{e}$	60 m	sensing radius of RSU nodes
speed	∼[5, 10] m/s	speed of nodes
data	50,500 KB	size of sensing data at LCS/HCS
cache_size	300 MB	cache size of nodes
e_length	∼[1000, 5000] s	life span of events
λ	200 s	interval of events (Poisson distribution)
*m*	3	number of readings at RSU for event-checking
Δ	0.05	predefined increment factor for $\tau_{1}$
TU	10,000 s	unit of time for threshold adjustment
$\epsilon_{l},\epsilon_{h}$	∼[0,0.5], ∼[0.0.1]	range of noise in LCS and HCS
$\sigma_{l},\sigma_{h}$	0.1, 0.05	standard deviation of weight in LCS and HCS

**Table 2:** Comparison of algorithms under default parameters.

Algorithm	NAIVE	PROPHET	ESSMD	TPEG
True Positive (#/ratio)	42.2/0.2497	147.62/0.8242	150.63/0.8389	147.47/0.8317
True Negative (#/ratio)	7.51/0.0444	17.32/0.0967	16.68/0.0929	14.66/0.0827
False Positive (#/ratio)	2.11/0.0125	3.47/0.0194	2.4/0.0134	2.81/0.0158
False Negative (#/ratio)	117.19/0.6934	10.69/0.0597	9.84/0.0548	12.38/0.0698
Recall Rate (*p*1)	0.2648	0.9325	0.9387	0.9226
Precision (*p*2)	0.9524	0.977	0.9843	0.9813
Transmissions ($\times 10^{4}$)	2.34	3421.98	2583.36	403.26
Average Time (minute)	36.29	28.83	30.6	33.69

**Table 3:** Performance with different incremental factors on $\tau_{1}$.

Incremental Factor (Δ)	0.03	0.05	0.07	0.1
Recall Rate (p1)	0.9554	0.9265	0.9091	0.8929
Precision (p2)	0.9350	0.9403	0.9459	0.9346
Transmissions ($10^{4}$)	618.77	578.38	556.78	532.22
Average Time (minute)	24.73	25.65	30.12	37.55
